# Oral Care Strategies to Suppress Salivary Bacterial Growth for the Prevention of Aspiration Pneumonia in Older Individuals Requiring Long-Term Care

**DOI:** 10.7759/cureus.90547

**Published:** 2025-08-20

**Authors:** Madoka Funahara, Yuki Sakamoto, Ryuichiro Funahara, Sakiko Soutome

**Affiliations:** 1 School of Oral Health Sciences, Faculty of Dentistry, Kyushu Dental University, Kitakyushu, JPN; 2 Department of Oral Surgery, Kansai Medical University Medical Center, Moriguchi, JPN; 3 Department of Oral Surgery, Funahara Dental Clinic, Kakogawa, JPN; 4 Department of Oral Health, Nagasaki University Graduate School of Biomedical Sciences, Nagasaki, JPN

**Keywords:** aspiration pneumonia, older adults requiring long-term care, oral care, oral function, salivary bacteria

## Abstract

Japan’s aging population faces a significant gap between life expectancy and healthy life expectancy, during which many older adults require long-term care and are at high risk of aspiration pneumonia, a leading cause of death. Aspiration pneumonia arises from the interaction of saliva-borne bacteria, aspiration events, and immune decline. Toothbrushing, while essential for oral health, may transiently increase bacterial counts, especially in those unable to rinse or gargle. Combining brushing with moisturizing gel and povidone-iodine gel effectively suppresses bacterial load, reducing pneumonia risk. Tailored oral care based on swallowing function, along with maintaining oral function through dental treatment, is crucial to prevent aspiration pneumonia in older adults requiring nursing care. This review highlights that increased salivary bacterial load, a key factor in pneumonia risk, is more strongly linked to reduced oral function (e.g., decreased tongue pressure and swallowing ability) than to poor oral hygiene alone.

## Introduction and background

Aspiration pneumonia is a major cause of morbidity and mortality among older adults worldwide, especially those with physical frailty or requiring long-term care. One key factor contributing to its development is poor oral hygiene, which increases the salivary bacterial load and the risk of infection following aspiration. As global populations age, preventive strategies that address modifiable risk factors such as oral bacterial burden are becoming increasingly important [1,2].

In Japan, the average life expectancy is 81.1 years for men and 87.1 years for women, making it one of the most rapidly aging societies in the world. In contrast, the “healthy life expectancy,” defined as the period during which individuals can perform activities of daily living (ADLs) independently and perceive themselves as being in good health, is 72.6 years for men and 75.5 years for women. This indicates a gap of more than 10 years between life expectancy and healthy life expectancy, during which many older adults require long-term care [3].

Aspiration pneumonia is a growing concern not only in Japan but also in other aging societies. For example, in the United States, pneumonia is among the leading causes of infection-related death in older adults, particularly among those residing in nursing homes [4-6]. A study in the United Kingdom reported that among cases of community-acquired pneumonia, those with aspiration risk are more common in older adults and are associated with higher mortality rates [7]. Similarly, in South Korea, another rapidly aging Organisation for Economic Co-operation and Development (OECD) country, the life expectancy in 2023 was approximately 83.5 years, while healthy life expectancy ranged from 66.3 to 71.8 years in recent years. This gap of more than 10 years, sometimes reaching up to 17 years, underscores a prolonged period in which older adults live with illness or functional impairments, paralleling the Japanese context and reinforcing the global relevance of investigating health span as well as lifespan [8].

Furthermore, several international studies have demonstrated that oral care interventions significantly reduce the incidence of aspiration pneumonia. A systematic review and meta-analysis by Barnes concluded that professional oral care reduced pneumonia risk in nursing home residents by up to 40% [4]. These findings underscore the importance of oral care in preventing aspiration pneumonia across various aging populations globally.

Pneumonia is the third leading cause of death among Japanese individuals aged 80 and above. Among these cases, aspiration pneumonia accounts for a large proportion of pneumonia-related deaths in older adults. Therefore, its prevention is a key issue in extending healthy life expectancy. According to national vital statistics, pneumonia consistently ranks among the top causes of death in adults aged 80 and above. For example, the proportion of deaths due to pneumonia was 11.4%, 14.0%, 15.9%, 16.5%, and 16.4% across the 80-84, 85-89, 90-94, 95-99, and 100+ age groups, respectively (average: 14.84%) [9]. Because aspiration pneumonia constitutes a major portion of pneumonia cases in older adults, its prevention is critically important for extending healthy life expectancy in aging populations.

Aspiration pneumonia develops through the interaction of three major risk factors: the presence of pathogenic microorganisms in saliva, the occurrence of aspiration, and decreased immune function. Consequently, in addition to nutritional support and frailty prevention, controlling the bacterial load in saliva is essential for reducing the risk of aspiration pneumonia.

In 2002, Yoneyama et al. [10] were the first to report that oral care interventions could help prevent aspiration pneumonia in older adults requiring nursing care. Their study implemented a multifaceted oral care regimen, including weekly plaque and calculus removal by dental hygienists, toothbrushing after every meal by caregivers, gargling with povidone-iodine, and direct application of povidone-iodine to the oral mucosa for those unable to gargle. Thus, “oral care” typically refers not to a single procedure but to a comprehensive approach involving multiple interventions. However, it remains essential to clarify which specific oral care practices are most effective in preventing pneumonia and to determine the target patient populations for these interventions.

As mentioned above, the pathogenesis of aspiration pneumonia involves multiple contributing factors, making the design of clinical studies aimed at identifying effective oral care methods inherently complex. To address this challenge, we have focused on one of the fundamental mechanisms of aspiration pneumonia: the bacterial load in saliva. Our research has aimed to identify the factors that contribute to increased salivary bacterial counts and to determine the most effective oral care techniques for reducing this bacterial burden. In this review, we provide an overview of our findings and discuss the optimal strategies for oral care in the prevention of aspiration pneumonia among older adults.

This article is a narrative review that summarizes our previous research findings alongside selected studies from the literature. It is not a systematic review and does not follow Preferred Reporting Items for Systematic Reviews and Meta-Analyses (PRISMA) or other formal guidelines.

## Review

Reports on oral factors related to pneumonia onset in older adults

One of the causes of aspiration pneumonia is an increase in the number of bacteria in saliva. Previous reports have indicated an association between dental plaque, periodontal disease, and salivary bacterial load. Tohara et al. [11] reported that a higher number of remaining teeth and the presence of food residue were significantly associated with an increased number of bacteria in the saliva of older adults requiring nursing care. Schaeken et al. [12] reported that *Actinomyces* and *Streptococcus mutans* in saliva increase in conjunction with plaque accumulation. Regarding the relationship between oral conditions and pneumonia, Terpenning et al. [13] and Son et al. [14] reported that an increase in dental caries raises the risk of aspiration pneumonia, and Awano et al. [15] found that a higher number of teeth with periodontal pockets was associated with a greater risk of pneumonia. These findings highlight the significance of controlling oral bacterial sources. In addition, Scannapieco and Shay emphasized that oral biofilm accumulation in dependent older adults is a major risk factor for aspiration pneumonia, reinforcing the need for both mechanical and chemical disruption of oral biofilms [16]. While these findings suggest that plaque and periodontal pockets are sources of oral bacteria contributing to increased pneumonia risk, conflicting reports also exist. Although the amount of plaque and number of bacteria in periodontal pockets decrease with fewer remaining teeth, Suma et al. [17], Son et al. [14], and Tsuneishi et al. [18] have reported that the risk of pneumonia increases as the number of teeth decreases.

Thus, there is no consensus as to whether the number of teeth, plaque, or the extent of periodontal pockets are definitive risk factors for pneumonia onset. This discrepancy may be explained by the fact that bacterial load in saliva is influenced not only by plaque and bacteria within periodontal pockets, but also by oral function, specifically the oral self-cleansing mechanism. Therefore, we classified participants based on their level of oral function and investigated the factors influencing salivary bacterial count in each group.

Factors associated with salivary bacterial count

In Healthy Individuals

Several studies have reported that bacteria present in dental plaque and periodontal pockets may contribute to the onset of aspiration pneumonia [6,19]. We first examined the relationship between the amount of plaque and salivary bacterial count. Healthy volunteers were instructed to stop brushing their teeth for two days to allow plaque accumulation, and their salivary bacterial counts were then measured. In this study, the high-plaque condition was defined as the state after refraining from toothbrushing for two days, allowing plaque to accumulate. The low-plaque condition was defined as the state after toothbrushing removed this accumulated plaque. Bacterial quantification was performed using real-time polymerase chain reaction (PCR) targeting the 16S rRNA gene with universal primers [20]. After plaque was removed by brushing, the salivary bacterial count was measured again to evaluate the relationship between plaque and bacterial load. Under high-plaque conditions, the average plaque control record (PCR) [21] was 52.2%, and the mean salivary bacterial count was 5.14 ± 0.24 log colony-forming unit (CFU)/mL. In contrast, under low-plaque conditions, the average PCR was 2.58%, and the bacterial count was 5.14 ± 0.42 log CFU/mL. No significant difference in salivary bacterial count was observed between the two conditions [22].

In Older Adults With Independent ADLs

We investigated 40 older adults with independent activities of daily living (ADLs) to identify factors associated with salivary bacterial load. Items examined included age, sex, smoking and alcohol habits, hypertension, diabetes, oral dryness, number of remaining teeth, Oral Hygiene Index-Debris Index (OHI-DI) [23], and tongue coating index [24]. Analysis revealed that individuals with fewer remaining teeth had significantly higher salivary bacterial counts, and those with poor oral hygiene also tended to have higher bacterial levels [22].

In Older Adults Requiring Care but Consuming a Regular Diet

We surveyed 59 residents of a care facility for older adults in relatively good health who consumed a regular diet to examine factors associated with salivary bacterial counts. The factors evaluated included sex, age, physical decline, grip strength, weight loss, fatigue, reduced activity level, number of teeth, functional tooth units (FTUs) [25], denture use, oral hygiene, tongue coating index, oral dryness, and tongue pressure. Results showed that poor oral hygiene and low tongue pressure were significantly associated with increased salivary bacterial counts [26].

In Older Adults With Impaired Swallowing Function

We assessed 112 frail older adults with dysphagia who required thickened diets or enteral nutrition via gastrostomy. The factors evaluated included age, sex, general health status, nutritional intake method, oral moisture level, ability to rinse the mouth, number of remaining teeth, plaque, denture cleanliness, tongue coating index [27], and bacterial load on the tongue dorsum. Due to cognitive decline, measuring tongue pressure was difficult in many cases. Analysis revealed that individuals with lower food consistency (e.g., on enteral nutrition) and those unable to rinse their mouths had significantly higher salivary bacterial counts [28]. However, no significant relationship was found between oral hygiene and salivary bacterial load in this group.

Relationship between oral hygiene/function and salivary bacterial load

The oral cavity is equipped with a self-cleansing mechanism through saliva secretion and swallowing, which helps maintain a stable salivary bacterial count. In healthy individuals, these functions operate efficiently, keeping bacterial counts stable even with high plaque levels [29]. However, with aging, saliva production and swallowing gradually decline, weakening the self-cleansing mechanism. Even among older adults with independent ADLs, decreased oral function is common, and if oral hygiene is poor, salivary bacterial load may increase [30,31], thereby increasing the risk of aspiration pneumonia [32]. In older adults with severely impaired swallowing function who cannot consume a regular diet, the self-cleansing mechanism is nearly lost, resulting in a dramatic increase in salivary bacterial count. Patients with gastrostomy or endotracheal intubation may have over 100 times the bacterial load compared to healthy individuals [33,34]. These findings suggest that salivary bacterial load is more strongly influenced by decreased oral function and reduced self-cleansing capacity than by oral hygiene status alone. Therefore, simply brushing the teeth may not be sufficient to control bacterial levels. Furthermore, a recent study demonstrated that decreased tongue pressure is significantly associated with dysphagia and reduced survival rates in older adults requiring long-term care, underscoring the clinical importance of maintaining oral function as part of infection control and general health management [35].

Relationship between teeth and oral function

Numerous studies have reported that tooth loss leads to declines in oral functions such as mastication [36]. Maintaining tongue pressure is important for preserving the oral self-cleansing mechanism and preventing increases in salivary bacterial load. We previously reported that tooth loss can lead to reduced tongue pressure, and that fixed prostheses such as bridges and implants may help prevent this decline [37]. Additionally, we recently found that dentures may improve occlusal force and masticatory efficiency, potentially preventing declines in tongue pressure [38]. However, these findings are based on cross-sectional studies, and longitudinal studies are needed to determine whether prosthodontic treatment can restore tongue pressure and reduce salivary bacterial counts. Although no prior studies reported a direct relationship between the number of remaining teeth and salivary bacterial count, our recent findings indicate that a decrease in tooth number leads to declines in tongue pressure and masticatory function, thereby weakening the self-cleansing mechanism and increasing salivary bacterial load [26].

In addition to dental treatment aimed at preserving teeth, chewing function can be maintained through non-invasive interventions such as masticatory muscle training, oral exercise programs, dietary modifications to include firmer foods, and the use of chewing gum for occlusal rehabilitation. Speech therapists may also play a role in oral function therapy, particularly in frail older adults.

Although tooth loss reduces the accumulation of dental plaque, it simultaneously impairs oral functions such as mastication and swallowing. This leads to a diminished washout effect of saliva, ultimately resulting in an increased salivary bacterial count.

Brushing methods to suppress bacterial counts in saliva

Toothbrushing is a common oral care method, primarily intended for removing dental plaque. However, brushing can temporarily increase the number of bacteria in saliva. This is because plaque that had adhered to tooth surfaces disperses into the oral cavity during brushing. In healthy individuals, this is not a problem, as rinsing the mouth quickly removes the dispersed bacteria. However, older adults who are unable to rinse effectively may not be able to eliminate these bacteria, increasing the risk of aspiration pneumonia. To prevent an increase in salivary bacteria during brushing, several techniques have been implemented in clinical settings. These include using gauze or suction devices during brushing to prevent the spread of plaque, performing oral wiping afterward, using moisturizing gels on the toothbrush to inhibit plaque dispersion, and applying disinfectants. Among these approaches, Suzuki et al. investigated the characteristics of gel-type oral moisturizers and reported that, in simulated oral care procedures, they required smaller amounts and were easier to apply compared to liquid types [39]. However, in our study, the combination of gauze, suction, and wiping has a low recovery rate for dispersed bacteria [40]. This is likely because it is particularly difficult to completely prevent plaque dispersion when brushing the mandibular teeth. Studies have shown that using gels can moderately suppress the spread of plaque-derived bacteria into the oral cavity, although salivary bacterial levels still increase compared to pre-brushing levels [41]. We also confirmed that brushing with gels has better plaque recovery than wiping alone, but it does not reduce salivary bacterial counts to pre-brushing levels [42]. Brushing with a toothbrush dipped in the disinfectant povidone-iodine can disinfect dispersed plaque-derived bacteria, thereby suppressing the increase in viable bacterial counts in saliva. However, when plaque levels are particularly high, the bactericidal effect of povidone-iodine is insufficient to completely prevent the increase in bacterial load.

**Figure 1:**
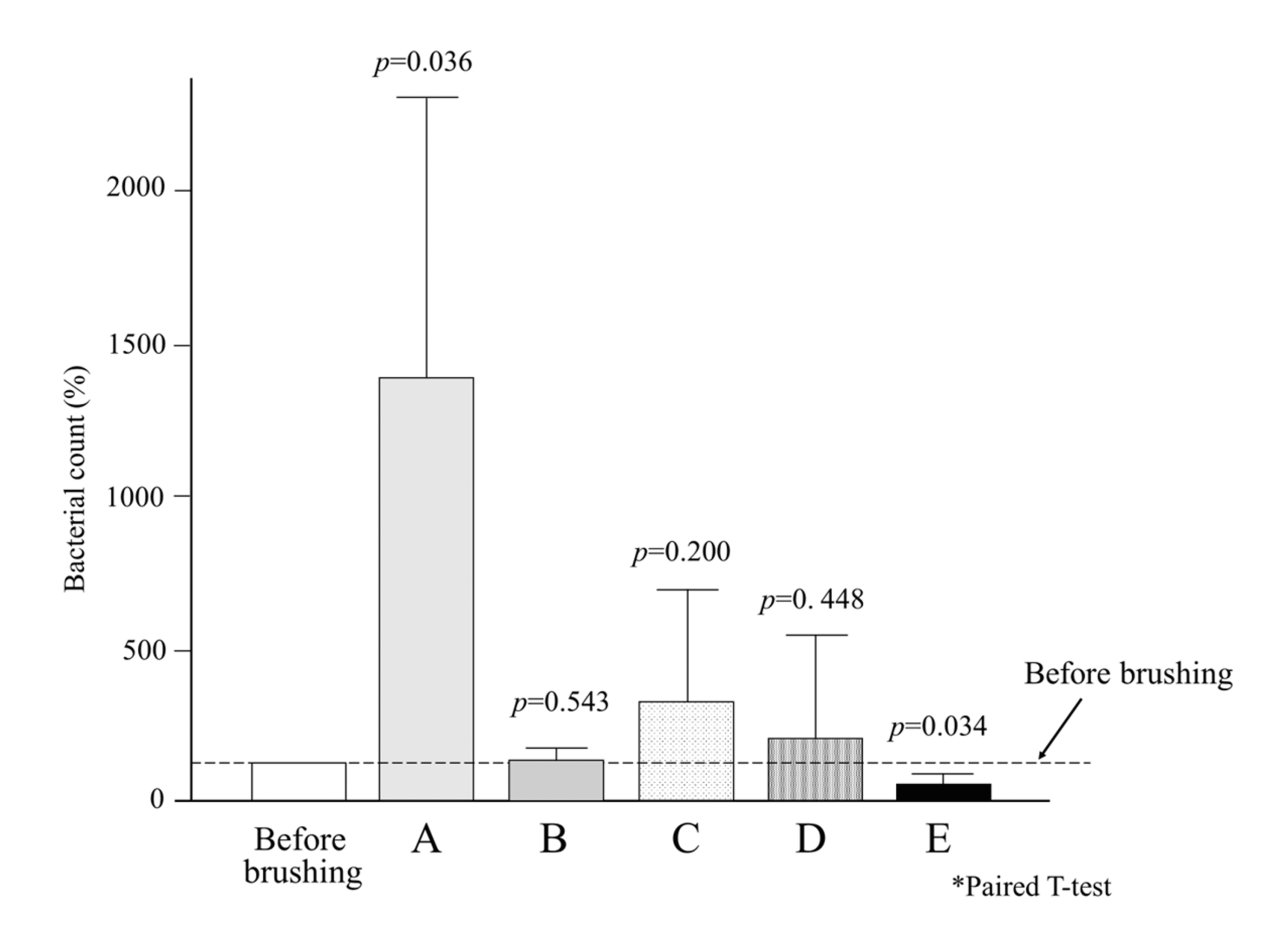
Changes in salivary bacterial count after brushing, expressed as a percentage relative to the pre-brushing baseline (set as 100) A: Salivary bacterial count after brushing with a toothbrush dipped in water. A significant increase in bacterial count was observed. B: Salivary bacterial count after rinsing following brushing. Rinsing returned the bacterial count to the pre-brushing level. C: Salivary bacterial count after brushing with a toothbrush coated with moisturizing gel. The spread of plaque was largely prevented, but the bacterial count remained higher than before brushing. D: Salivary bacterial count after brushing with a toothbrush dipped in 7% povidone-iodine solution. The increase in bacterial count was substantially suppressed, although still slightly higher than the pre-brushing level. E: Salivary bacterial count after brushing with a toothbrush coated with a mixture of moisturizing gel and povidone-iodine gel. A significant reduction in bacterial count compared to pre-brushing levels was observed. Image credit: Madoka Funahara

To leverage both the physical effect of moisturizing gel in preventing plaque dispersion and the chemical disinfectant action of povidone-iodine, we prepared a 9:1 mixture of moisturizing gel and iodine gel. When this mixture was applied to a toothbrush and used for brushing, it successfully prevented the spread of plaque and even reduced salivary bacterial levels below pre-brushing levels [42]. Figure 1 summarizes the data we have reported thus far. Brushing increases salivary bacterial counts, which could raise the risk of aspiration pneumonia. Rinsing can return the bacterial count to pre-brushing levels. However, using a mixture of moisturizing and povidone-iodine gel for brushing effectively suppresses the increase in salivary bacteria. Therefore, this method is recommended when providing oral care for older adults who cannot rinse their mouths.

Povidone-iodine is generally considered to have a high safety profile, although there is a rare risk of iodine hypersensitivity. Unlike other iodine-based preparations, it does not cause mucosal irritation. However, it is important to monitor for potential adverse effects during use.

Further improvements

**Figure 2:**
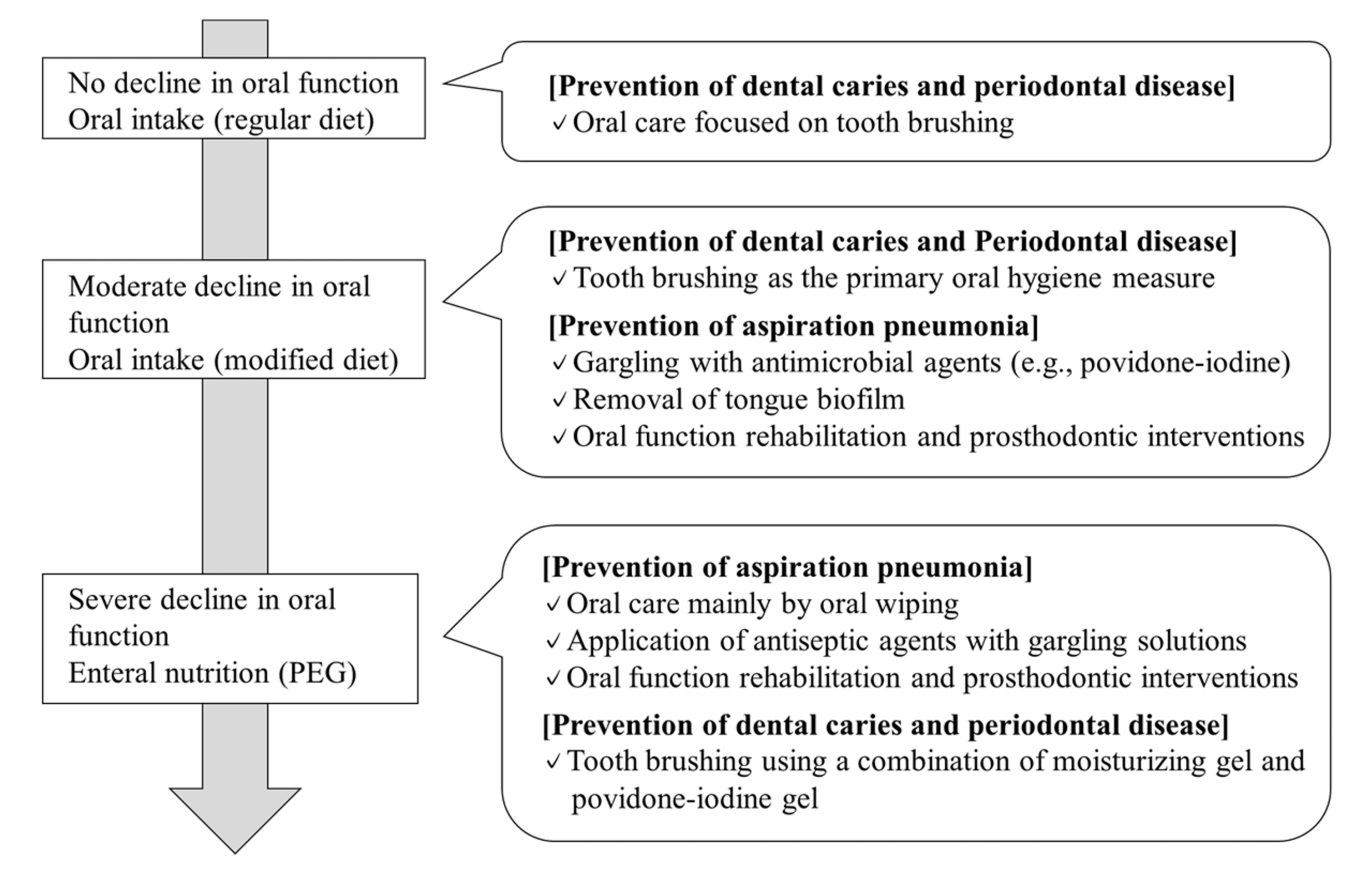
Oral care strategies considering the oral functional capacity of older adults Rather than emphasizing toothbrushing alone, oral care should be implemented with a focus on preventing aspiration pneumonia. With the progression of oral functional decline, the use of disinfectants such as povidone-iodine becomes an essential component of care. PEG: percutaneous endoscopic gastrostomy Image credit: Madoka Funahara

Based on our findings, we recommend that oral care for older adults be tailored according to their level of swallowing function (Figure 2). For older adults with normal swallowing function and a regular diet, regular brushing for the prevention of caries and periodontal disease is sufficient. In older adults with mildly impaired swallowing who consume thickened or puréed foods, the risk of aspiration and bacterial increase is elevated, so in addition to brushing, gargling with disinfectants such as povidone-iodine is recommended. In older adults with severely impaired swallowing who are on enteral feeding or cannot rinse their mouths, oral self-cleansing function is severely compromised, and salivary bacterial counts increase significantly. Brushing using a mixture of povidone-iodine gel and moisturizing gel is recommended. Moreover, to prevent aspiration pneumonia, it is more important to maintain oral function than to focus solely on plaque removal. Receiving regular dental treatment and preserving masticatory function are essential.

While previous studies have linked oral hygiene markers to pneumonia risk, their findings are inconsistent and often fail to account for oral function [43,44]. Furthermore, few studies have examined the independent impact of total bacterial load versus microbial composition. These gaps highlight the need for further research focused on the interaction between oral function, hygiene, and salivary microbiota in aspiration pneumonia development. These findings suggest that future studies should explore not only quantitative bacterial reduction but also the microbial profile and its relationship with oral function and systemic outcomes.

## Conclusions

In older adults requiring nursing care, reducing the number of bacteria in saliva through effective oral care is crucial for minimizing the risk of aspiration pneumonia. An increase in bacterial load is more strongly influenced by the loss of oral self-cleansing function due to declining oral function than by poor oral hygiene alone. Tooth loss contributes to a decline in tongue pressure, which in turn may lead to an increase in salivary bacterial counts. Whether prosthodontic treatment can restore tongue pressure and reduce bacterial counts remains a question that future longitudinal studies need to clarify. In older adults who are unable to rinse their mouths, toothbrushing may paradoxically increase the number of bacteria in saliva, thereby raising the risk of aspiration pneumonia. However, brushing with a mixture of moisturizing gel and povidone-iodine gel significantly reduces bacterial counts. We plan to conduct a large-scale clinical study to evaluate the effectiveness of prosthodontic treatment and this novel oral care method.

One limitation of this study is that we did not assess the specific types or pathogenicity of the oral bacteria. Future studies using microbiome analysis or culture-based methods could provide further insights into the relationship between bacterial composition and aspiration pneumonia risk.
